# Molecular Relationship between Strains of *M*. *bovis* from Mexico and Those from Countries with Free Trade of Cattle with Mexico

**DOI:** 10.1371/journal.pone.0155207

**Published:** 2016-05-12

**Authors:** Feliciano Milian-Suazo, Leticia Garcia-Casanova, Suelee Robbe-Austerman, Germinal Jorge Canto-Alarcon, Isabel Barcenas-Reyes, Tod Stuber, Elba Rodriguez-Hernandez, Susana Flores-Villalva

**Affiliations:** 1 Facultad de Ciencias Naturales-Universidad Autónoma de Querétaro, Querétaro, Querétaro, México; 2 National Veterinary Services Laboratory-APHIS, United States Department of Agriculture, Ames, Iowa, United States of America; 3 CENID-Fisiología y Mejoramiento Animal-INIFAP, Colón, Querétaro, Querétaro, México; University of Padova, Medical School, ITALY

## Abstract

The purpose of this study was to identify relationships between spoligotypes of *M*. *bovis* from cattle in Mexico and those reported in countries with free trade of cattle with Mexico: Australia, Canada, New Zealand and the United States of America. Mexican spoligotypes were obtained from isolates collected from cattle in different parts of the country. Spoligotypes from Canada and New Zealand were obtained from different reports in the literature. Those from the United States were obtained from the database of the National Veterinary Services Laboratory in APHIS-USDA. In order to perform the analysis in a single data set, spoligotypes were all converted to binary data and classified according to www.mbovis.org or www.pasteur-guadeloupe.fr:8081. Epidemiologic information included country and species infected. From 3,198 isolates, 174 different spoligotypes were obtained, 95 were orphans. Ninety one percent of the isolates came from the Unites States (n = 1,609) and Mexico (n = 1,323). Spoligotype SB0265 is shared between Canada and the United States in cattle and wildlife. Six spoligotypes, SB0673, SB0121, SB0145, SB0971, SB0140 and SB1165, were frequent in cattle and wildlife in the United States and cattle in Mexico, suggesting wide exchange of strains. Spoligotype SB0669 was found only in Mexico. Spoligotype SB0140 was the most common in Australia and the sixth in the United States and Mexico. In a phylogenetic analysis, spoligotype SB0140 appears as the oldest spoligotype in the data set, suggesting this as the ancestral spoligotype for all spoligotypes in the five countries. Some spoligotypes are shared by animals and humans, corroborating the zoonotic importance of *M*. *bovis*.

## Introduction

*Mycobacterium bovis* is the cause of tuberculosis in cattle and a risk to public health [1]. The proportion of cases due to *M*. *bovis* in humans has been estimated in 0.5% to 7.2% [2, 3]. *M*. *bovis* is also responsible for severe damage to the livestock and wildlife industries. Therefore, eradicating *M*. *bovis* from cattle and wildlife is of significant importance to reduce risk to public health, to protect the livestock industry, and encourage the international trade of animals and animals’ products between countries [4].

Understanding the epidemiology of *M*. *bovis* has been enormously improved with the arrival of molecular methods [5–7]. Restriction fragment length polymorphisms (RFLP) with different target sequences, IS6110, PGRS and DR, spacer oligonucleotide typing (spoligotyping), mycobacterial interspersed repetitive units-variable number of tandem repeats (MIRU-VNTR) and single nucleotide polymorphisms (SNPs) are, worldwide, the most popular. However, due to simplicity, spoligotyping is perhaps the most widely used method for tracing back sources of infection and establish genetic relationships between strains.

Spoligotyping detects polymorphism within the genomic direct locus [6]. This chromosomal region contains a large number of direct repeats (DRs) of 36 bp interspersed with spacer DNA (DVRs) 35–41 bp in length. When the DR regions of several isolates are compared, it is observed that the order of the spacers is about the same in all isolates, but deletions and insertions within the DVRs occur. The polymorphisms in various isolates comprise the absence or presence of one or more DVRs, a characteristic that is used to determine genetic similarity of strains. In spoligotyping, the DR region is amplified by PCR with a single pair of primers, followed by hybridization of the labeled PCR product to immobilized spacer oligonucleotides. Strains with the same spoligotype are assumed to be individuals recently derived by clonal replication from a single ancestral cell [8]; therefore, epidemiological related strains should have higher genetic similarity than not related strains [9]. This is why spoligotyping is used to trace back sources of infection and to better understand the epidemiology of tuberculosis in different countries [10–23]; however, there is no much information about the application of this method to the study the relationships between strains of *M*. *bovis* from countries with a long history of animals exchange due to commercial agreements.

Bacilli of the *M*. *tuberculosis* complex are clonal, the exchange of genetic material between individuals is null [24– 26]. Thus, spoligotypes have enough phylogenetic information to estimate recent evolutionary events [25]; strains with the same spoligotype are assumed to be individuals recently derived by clonal replication from a single ancestral cell [8]. Because of this, there is a certain level of confidence in performing epidemiological studies.

In Mexico there is a national program to control and eradicate TB from cattle. This program has been successful in beef cattle, the prevalence has been reduced to <0.5% in 85% of the national territory (Plan Estratégico para el Control de la Tuberculosis Bovina, SENASICA-SAGARPA, 2008–2012) [24]. However, there are spots in dairy areas were the prevalence is high, making difficult to accomplish the program goals; external sources of infection through exchange of cattle could at least partially explain the persistence of the disease. Therefore, the purpose of this study was to determine the relationship between *M*. *bovis* strains from cattle in Mexico and those from countries with history of free trade of cattle with Mexico. Mexico has a long history of exchange of cattle with different countries. It exports an average of 1.4 million calves a year to the United States of America (USA) for fattening, and imports replacements for milk production from USA, Canada, Australia and New Zealand.

## Materials and Methods

### Mycobacterial isolates and strains from Mexico

One-thousand three-hundred and twenty-three *M*. *bovis* isolates obtained between 2003 and 2010 from cattle from different regions of Mexico were included in the study: 269 came from banks of isolates in regional TB-diagnostic laboratories around the country, and 1,054 were obtained by culturing TB-suspicious lesions collected at slaughterhouses and some from reports in the literature. This was not a representative sample, however, it reflects the prevalence of the disease in the states from were isolates could be collected; 26 out of 32 states were represented. The protocol of this project was approved by the Bioethics Committee of the School of Natural Sciences of the Autonomous University of Queretaro. S1 Table

### Mycobacterial isolates and strains from other countries

Spoligotypes from countries with free trade of cattle with Mexico were obtained from different sources in the literature. The year considered was that of the publication: 216 from Australia reported in 1998, 69 from Canada, also reported in 1998, 1,425 from the USA reported in 2008 but collected along the years since the early 90’s, and one from New Zealand. In order to perform the spoligotypes comparison, spoligotypes were all converted to binary data and classified according to www.mbovis.org or www.pasteur-guadeloupe.fr:8081. Epidemiologic information included country and affected species. S1 Table

### Molecular analysis protocol

According to the reports, spoligotyping was performed following Kamerbeek’s [6] protocol. Briefly, the DR region was amplified using the primers DRa (GGTTTTGGGTCTGACGAC, 5’ biotinylated) and DRb (CCGAGAGGGGACGGAAAC). The amplified product was hybridized to a nylon membrane to which 37 spacer sequences from *M*. *tuberculosis* H37Rv and 6 spacer sequences from *M*. *bovis* BCG were covalently bound (Isogen Bioscience BV, Maarsen, the Netherlands). For the detection of hybridizing DNA, chemiluminescent ECL detection liquid (Amersham Biosciences; Pistacaway, NJ) was used, followed by exposure to X-ray film (Kodak) for 45 min.

### Phylogenetic analysis

Spoligotypes were converted to discrete character data, 0 (absent) and 1 (present), for all 43 probe hybridization positions. Relationship among spoligotypes was established using the SPOLIGOFOREST program in the spolTools webpage (www.emi.unsw.edu.au/spoltools/) [26] for all most frequent spoligotypes in each country. Spoligoforest provides a visualization of the probable relationships among spoligotypes in a given sample [27]. The method makes use of a model that considers mutation by irreversible deletions of spacers and assigns probabilities to the lengths of these deletions. The size of each node is an increasing function of the number of isolates (i.e., the cluster size); edges between nodes reflect evolutionary relationships between spoligotypes with arrowheads pointing to descendants. To determine emerging strains, strains that are spreading faster than the background rate, the recent transmission index (RTI) was estimated with DESTUS [28]. The RTI measures the extent of recent tuberculosis transmission, and is usually expressed as the proportion of clustered cases. It is estimated as: RTI = n − g/n − 1, where **n** is the sample size and **g** the number of distinct genotypes in the sample.

## Results

A total of 3,198 isolates of *M*. *bovis* were included in the study, the most frequent ones for each country in the database are shown in table 1. All isolates grouped into 269 different spoligotypes, 174 with at least 2 isolates and 95 orphans. Ninety one percent of all isolates came from the Unites States (n = 1,609) and Mexico (n = 1,323).

**Table 1:** Most Frequent spoligotypes of *M*. *bovis* from Mexico and countries with history of free trade of cattle with Mexico.

Country	Spoligotype	Frequency*	Percentage**	Species (n)
**New Zealand**	SB0130	1	100	Possum
**Subtotal**		1		
**Canada**	SB 0265	36	52	Cattle(26), Elk(3), Yak(1), bison(5), human(1)
	SB1069	18	26	Cattle(10), Elk(2), deer(6)
	SB0337	6	8.7	Cattle(6)
	SB1070	2	2.9	Cattle(2)
	SB1071	2	2.9	Cattle(2)
**Subtotal**		**64**		
**Australia**	SB0140	152	70	Cattle(152)
	SB1030	27	12	Cattle(27)
	SB0289	7	3.2	Cattle(7)
	SB0273	6	2.7	Cattle(6)
	SB0144	5	2.3	Cattle(5)
	SB1031	4	1.8	Cattle(4)
**Subtotal**		**201**		
**United States**	SB0145	613	38	Cattle(260),Wildlife (299), cattle from Mexico(23), feral pig(13), unknown(9), farmdeer(7), exotic(2).
	SB0673	281	17.5	Cattle USA(142), cattle from Mexico(101), cattle from Canada(3), unknown(25), human(10)
	SB0265	146	9	Cervid(116), elk(1), cattle(29)
	SB2011	109	6.7	Cattle(109),
	SB0271	94	5.8	Cattle(55), Wildlife(39).
	SB0140	65	4	Cattle from Mexico(32), Zoo animals(12), unknown origin cattle(11), USA cattle(7), human(3)
	SB0971	46	2.8	Mexican origin cattle(32), USA Cattle(10), unknown origin cattle(4)
	SB0121	27	1.7	Cattle(17), Zoo deer(3), wildlife deer(1), cattle unknown origin(6)
	SB0327	25	1.5	Mexican origin cattle(17), USA cattle(5), Unknown origin cattle(3)
	SB1040	21	1.3	Human(14), Mexican origin cattle(5), unknown origin cattle(2)
	Other	182	11.3	
**Subtotal**		**1,609**		
**Mexico**	SB0673	155	11.7	Cattle(155), human(2),
	SB0669	124	9.4	Cattle(122), human(2)
	SB0121	81	6.1	Cattle(80), human(1)
	SB0145	78	5.9	Cattle(76), human(2)
	SB0971	70	5.3	Cattle(69), human(1)
	SB0140	71	5.3	Cattle(66), human(5)
	SB0663	50	3.8	Cattle(49), human(1)
	SB0269	42	3.1	Cattle(44)
	SB1116	42	3.1	Cattle(42)
	SB0120	29	2.2	Cattle(29), human(1)
	SB0119	25	1.9	Cattle(24), human(1)
	SB1165	13	1	Cattle(10)
	Other	543	41	
**Subtotal**		**1,323**		
**TOTAL**		**3,198**		

*Not all spoligotypes are listed.

**Proportion from all spoligotypes analyzed.

Two spoligotypes were by far the most frequent ones in Canada, SB0265 and SB1069, found in cattle and wildlife, suggesting transmission between these two species. Spoligotype SB0265 is shared with the United States. In both countries most isolates with this spoligotype come from wildlife, clearly indicating a common epidemiological problem for these two countries. Spoligotype SB1069, the second most frequent in Canada was not frequent in any of the other countries.

The most frequent spoligotype in Australia was SB0140, isolated only from cattle. This spoligotype is the 6^th^ most frequent one in the USA, found in cattle, Zoo animals and humans; and the 6^th^ most frequent one in Mexico, isolated from cattle and humans.

One-thousand six hundred and nine isolates were reported from the USA, 77% grouped into only five spoligotypes: SB0145, SB0265, SB0271, SB2011 and SB0673. Spoligotypes SB0145, SB0265, SB0271 are shared between cattle and wildlife, indicating a serious epidemiological problem for this country, since eliminating tuberculosis from wildlife is much more complicated than eliminating it from cattle. Spoligotypes SB2011, SB0673 and SB0140, the 4^th^, 5^th^ and 6^th^ most common spoligotypes were found only in cattle. Interestingly, three spoligotypes, SB0673, SB0140 and SB1040 were found in isolates from cattle and humans, corroborating the zoonotic importance of *M*. *bovis*.

In the case of Mexico, 1,323 isolates were obtained, grouped into 59 spoligotypes with at least 2 isolates and 94 orphans. Twelve of the spoligotypes had at least 13 isolates, and included 61% of the total isolates; two were the most frequent, SB0673 (n = 155 isolates) and SB0669 (n = 124), which accounted for 21% of all isolates in the country. Most of the spoligotypes were reported in cattle and a few in humans, once more, confirming transmission of tuberculosis from cattle to humans. No spoligotypes have been, up to now, reported in wildlife in Mexico. Only one spoligotype was reported in a brushtail possum in New Zealand.

The most important share of spoligotypes occurred between Canada and the Unites States, and between the United States and Mexico. Canada and the United States share spoligotype SB0265, most of the isolates coming from wildlife, stressing the well known problem of tuberculosis in wildlife of these countries in the Ontario- Michigan border. The United States and Mexico share four of the most common spoligotypes in the United States, SB0145, SB0673, SB1040 and SB0971. SB0145, the most frequent spoligotype in the US, is the 4^th^ most common in Mexico. In the US this spoligotype has about the same frequency in cattle (n = 260) than in wildlife (n = 299), while in Mexico has only been isolated from cattle. Spoligotype SB0673, the most common spoligotype in Mexico, is the 2^nd^ most common in the US. In both countries this spoligotype has been isolated only from cattle. This is explained by the large history of animals exchange by these two countries. Interestingly, spoligotype SB0669, the second most common in Mexico, seems to be exclusive of this country, only two isolates from the USA had this spoligotype.

Spoligotype SB0140 was the most common spoligotype in Australia, the 6th most common in the USA and Mexico. In a phylogenetic analysis of all isolates, SB0140 appears as the oldest spoligotype in the data set, suggesting this as the ancestral for all spoligotypes in the four countries.

**Fig 1:**
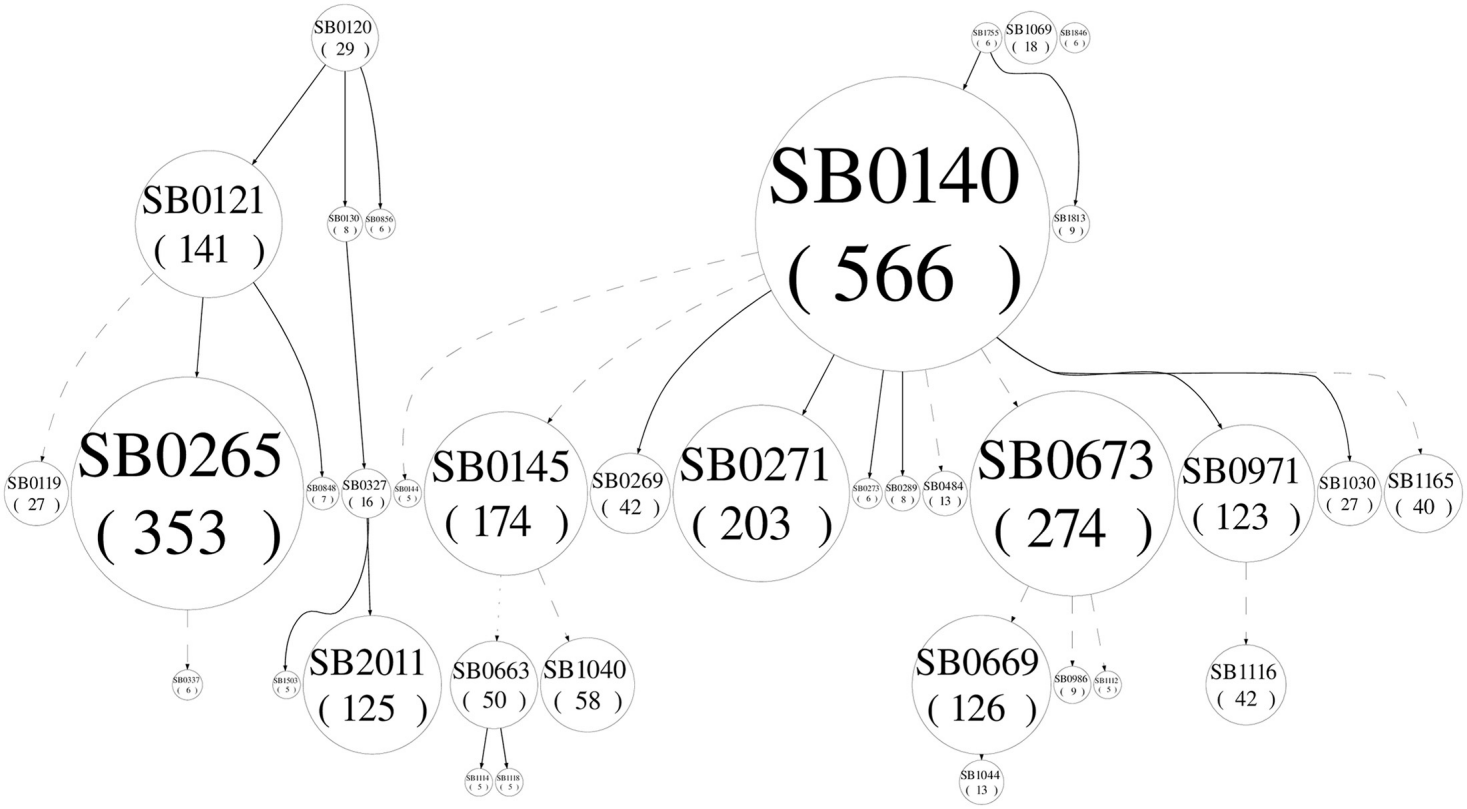
Spoligoforest of *M*. *bovis* spoligotypes obtained from cattle in Mexico and those from countries with history of trade of cattle with Mexico: Australia, Canada, New Zealand and The Unites States of America. Nodes are labeled with the SB identifier as indicated in www.mbovis.org; numbers in the circle indicate cluster size. The lines between nodes reflect hypothetical evolutionary relationships among spoligotypes with arrows denoting descendence. Continuous lines indicate stronger relationship.

A phylogenetic analysis was performed in the dataset. Fig 1 shows the spoligoforest hierarchical layout where the continuity of lines indicates the weight of the hypothetical evolutionary relationship between spoligotypes; continuous line indicates stronger relationship. The spoligoforest shows two trees with connected components. The biggest tree, the one with the largest number of branches, is rooted by strain SB0140; suggesting this as the oldest strain in the dataset. A large number of spoligotypes descend from SB0140, four of which are in large clusters (range 123–274), and seven in small clusters (range 5–42). The hypothetical evolutionary relationship between spoligotypes SB0140 and two of the four large groups (SB0271 and SB0971) are connected by continuous lines, suggesting direct evolutionary pathway. Two of these clusters (SB0145 and (SB0673) are connected by dotted lines, suggesting intermediate steps in the evolution process. Five of the seven small clusters are connected by continuous lines with strain SB0140, indicating direct evolutionary pathway and suggesting that this strain is still successful in generating new genetic lines. Spoligotype SB0669, a spoligotype almost exclusive to Mexico, indirectly descends from spoligotype SB0673, a frequent spoligotype in both Mexico and USA, suggesting a large number of changes in the sequence of the direct repeat region, assuming that there is an evolutionary relationship between them. Likewise, spoligotype SB0669, the second largest cluster in the Mexican dataset, has a weak relationship with spoligotype SB0673, and is only indirectly related to spoligotype SB0140.

The smallest tree is rooted by spoligotype SB0120, suggesting this as the oldest strain in this tree. Tree spoligotypes directly descend from SB0120, however, spoligotype SB0121 has the largest number of isolates, suggesting more success of this spoligotype in spreading. A spoligotype with a large number of isolates directly descends from spoligotype SB0121.

## Discussion

The relationship of *M*. *bovis* strains from countries with long history of cattle exchange has been searched in order to better understand the role of commercial activities in the dissemination of TB. Our results suggests that this practice is a plausible mechanism of tuberculosis dissemination between countries.

The most important share of spoligotypes occurred between Canada and the USA, and between the USA and Mexico. Sharing of strains between Canada and the USA can be explained for the well documented problem of tuberculosis in wildlife in the Ontario-Michigan border [29]; since 1994 the state of Michigan has been experiencing outbreaks of tuberculosis in wild deer, some of these deer may be moving back and forth across the Canadian-USA border making this a mutual wildlife problem.

In the case of the USA and Mexico, sharing of strains may be a consequence of the long history of cattle exchange. An average of 1.4 million calves cross the Mexican-USA border every year for fattening in the US, and the USA exports an average of 2,700 heifers every year to Mexico as replacements for dairy; except from 2003 to 2006, when the Mexican border was closed due to the bovine spongiform encephalopathy outbreak in the USA. No spoligotypes are common to Mexico and Canada in spite of the continuous movement of dairy cattle from Canada to Mexico every year. Exportation from Mexico to Canada is not common.

SB0140 was the most frequent spoligotype in Australia, and the sixth most frequent one in the USA and Mexico. SB0140 has been identified as an important member of a globally important clonal complex of *Mycobacterium bovis*, the European 1 clonal complex (Eu1) [30]. The hypothetical origin of this spoligotype is in the UK, which could have been taken to the British Islands long time ago. Strains of the Eu1 clonal complex are identified by the loss of spacer 11 in the spoligotype pattern and the deletion of the RDEu1 region, an 806 bp deletion region of chromosomal DNA. The fact that most of the spoligotypes in our data set lack spacer 11, a characteristic of the Eu1 complex, supports the hypothesis that the most frequent spoligotypes in Mexico are part of the Eu1 complex and derive from the SB0140 spoligotype; therefore, SB0140 could have been imported to Mexico with dairy cattle. As a matter of fact, SB0140 appears as the oldest spoligotype in the whole data set in the spoligoforest tree (Fig 1), suggesting this as the ancestor of all spoligotypes in the five countries. SB0140 has been found to be the most frequent spoligotype infecting cattle, deer, badgers [31] and people [2] in Ireland, cattle in the United Kingdom [31, 32], pigs [33], cattle and cats in Argentina [34], and humans in the United States [35].

SB0130 was the only spoligotype reported in New Zealand, no other of the five countries had the same spoligotype. In spite of the problems of TB in the brushtail possum, New Zealand has a very low prevalence of TB in cattle [36].

Highly prevalent genotypes associated with outbreaks are considered “fast spreading”; therefore, strains (in this case defined as a group of isolates with the same spoligotype), that are spreading faster than the background rate are considered “emerging strains.” The approach to identify emerging strains uses information of the mutation process specific to spoligotypes combined with a model of both transmission and mutation. The hypothesis is that if two strains have the same number of isolates, then the strain with fewer mutation events must have spread faster if the mutation process is common [37]. Emerging strains are identified throughout the recent transmission index (RTI). In this data set the RTI was high (0.98), indicating active transmission of TB in the population; however, SB0265 was the only strain with a significant transmission rate (q-value = 0.000774), suggesting this as a very active and successful strain in generating new outbreaks of the disease. The reason about why this strain is identified as an emerging strain it is not clear, it could be because there are not barriers to transmission of the host population; SB0265 has been isolated from cattle and wildlife in the border between Canada and the USA.

SB0140 is a strain with a large number of isolates (n = 566); however, it was not identified as an emerging strain. In general, a large cluster size is attributable to the age of the strain rather than to rapid transmission [37], this agrees with the result from the spoligoforest analysis, which identifies SB0140 as the ancestral strain in the dataset.

The hypothesis of our study is that presence of a specific spoligotype in different country is a consequence of the exchange of animals along the years; however, the probability of convergent evolution, also known as homoplasy, of the DR marker is possible; similar mutations in cattle from different countries could have arisen from phylogenetically unrelated strains. However, given the clonal structure of *M*. *bovis*, the unidirectionality of mutations (spacers can only be lost not re-acquired), and that the average genetic stability of the DR marker has been estimated in between 10 and 20 years [38,39], support our conclusions.

In most countries beef and dairy farming is performed under different conditions; while dairy farming is done in intensive conditions with high population densities, beef farming is done in more extensive areas with a lower number of cattle per unit area; this plays a role in the dissemination of tuberculosis, for different reasons, the prevalence of the disease is higher in dairy cattle. The exchange of animals between countries in our study involves mainly dairy cattle; however, the exchange of animals between Mexico and the US involve both, dairy and beef cattle. Most isolates from Mexico come from dairy cattle, reflecting the situation of tuberculosis in cattle in the country. Isolates from the US come from beef and dairy cattle, and from wildlife. The exchange of *M*. *bovis* strains between dairy and beef cattle is believed to happen during the fattening period, when Mexican and US calves are mixed in the same corrals during the fattening period, about one year. Infection from beef to dairy cattle within the USA may be happening throughout wildlife. It is common to see white-tail deer in the same pastures with both, beef and dairy cattle.

## Conclusions

There are spoligotypes of *M*. *bovis* with a large number of isolates that are shared between countries, suggesting exchange of strains, probably throughout exportation or importation of cattle and close contact between populations, as is the case of wildlife between Canada and the US. There are, however, some strains that have been able to adapt to specific populations, for example, spoligotype SB0669, present only in Mexico. SB0140 is the most common spoligotype in many countries, and seems to be the ancestral clone of all spoligotypes in the countries included in this study. SB0265 was the only strain with a significant transmission rate (q-value = 0.000774), suggesting new outbreaks in the population where this strain is present. Finally, it has been shown that spoligotyping is a useful tool to better understand the epidemiology of *M*. *bovis* around the world.

## Supporting Information

S1 Table Spoligotypes of *M*. *bovis* from Mexico and countries with history of free trade of cattle with Mexico analyzed in this study. (XLSX)
